# Life‐history evolution in the orange‐tailed skink populations living in different climates

**DOI:** 10.1002/ece3.11521

**Published:** 2024-06-18

**Authors:** Abdullah Altunışık, Mehmet Zülfü Yıldız, Hatice Hale Tatlı, Deniz Yalçınkaya, Bahadır Akman

**Affiliations:** ^1^ Biology Department, Faculty of Arts and Sciences University of Recep Tayyip Erdoğan Rize Türkiye; ^2^ Zoology Section, Biology Department, Faculty of Arts and Sciences Adıyaman University Adıyaman Türkiye; ^3^ Medical Laboratory Techniques Program, Department of Medical Services and Techniques, Vocational School Toros University Mersin Türkiye; ^4^ Technical Sciences Vocational School Iğdır University Iğdır Türkiye

**Keywords:** age, growth, sexual dimorphism, skeletochronology, survival rate

## Abstract

The life‐history traits of ectothermic animals can be influenced by many abiotic factors, including climate. As an ectothermic species, we questioned whether the life‐history characteristics of the orange‐tailed skink (*Eumeces schneiderii*) populations differ between two different environments/climates. Our findings showed that the average body size of lizards living in the Mediterranean climate zone was higher than those in the continental climate zone. However, although Mediterranean population had higher mean values regarding average age, there was no discernible difference between the two climate zone populations. When considering all populations collectively, it has been discovered that the species' maximum lifespan is 18 years. Body size notably increased with age in both populations. Through the utilization of the von Bertalanffy equation, the anticipated growth parameters portrayed a highly accurate connection between age and snout–vent length. In conclusion, lizards living in habitats characterized by milder Mediterranean climates were found to have larger body sizes than continental populations, but both populations were comparable in terms of mean age. This difference can be explained by several factors, including activation time, temperature, precipitation, food abundance, and the presence of predators.

## Graphical abstract

The life‐history traits of ectothermic animals can be influenced by many abiotic factors, including climate. As an ectothermic species, we questioned whether the life‐history characteristics of the orange‐tailed skink (*Eumeces schneiderii*) populations differ between two different environments/climates. Our findings showed that lizards living in habitats characterized by milder Mediterranean climates had larger body sizes than continental populations, but both populations were similar in terms of mean age.

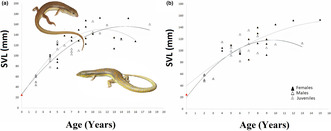

## 1 INTRODUCTION

Climate‐related parameters such as temperature, precipitation, and photoperiod can affect the life history of ectothermic animals (Cabezas‐Cartes et al., 2018; Shine, 2005). Some life‐history traits such as age at sexual maturity, age structure, growth rate, and sexual dimorphism are among the most important parameters that show eco‐geographical variation among species and populations (Altunışık, Yıldız, et al., 2022; Cruz‐Elizalde et al., 2020; Dubey et al., 2013). For instance, at high latitudes and altitudes where temperatures are lower, lizards have short activity seasons, and populations are characterized by slow growth rate, delayed sexual maturity, and extended lifespan (Kubisch et al., 2012; Piantoni et al., 2006). Longevity is influenced by intrinsic factors such as pleiotropic genes (Ljubuncic & Reznick, 2009) and the formation of toxic metabolic waste products (Sohal, 1986; Wilkinson & South, 2002), as well as extrinsic factors such as mortality from hunting or disease (Cabezas‐Cartes et al., 2018).

Estimating longevity and growth are two critical life‐history parameters that are necessary to define a species' life history, which is crucial for effective conservation efforts. While determining the age of animals can be challenging, skeletochronology provides a trustworthy technique for calculating age in ectothermic species such as lizards. Within ectothermic species, variations in bone tissue formation, influenced by climatic conditions, serve as indicators for estimating an individual's age in the wild. This alteration and histomorphological structure provide insights into the individual's growth, survival rate, life expectancy, and sexual maturity. Lizards are ideal model organisms for studying parameters related to demography and body size (Sabath et al., 2016). In this context, the life‐history characteristics of lizard populations living in different climates can be revealed by the skeletochronology, enabling comparison of the data (Altunışık & Eksilmez, 2018).

Environmental conditions such as temperature variations between habitats and variations in food quantity and quality can significantly induce stress and exert a profound impact on individual life histories (Adolph & Porter, 1993; Mesquita & Colli, 2010; Reniers et al., 2015). These effects manifest in various ways, affecting parameters like growth rate, age of maturation, body size, clutch/pup size, and other characteristics (Mesquita et al., 2016). Consequently, at the population level, the environment's impact on physical development can be observed (Mugabo et al., 2010). Variations in ambient temperature have short‐term effects on an individual's body temperature (González‐Morales et al., 2021; Sears, 2005; Tucker, 1966), as well as long‐term effects on key life‐history traits such as growth, survival, and reproduction (Adolph & Porter, 1996; Lu et al., 2018). For instance, lizards living at high altitudes in temperate regions undergo more significant thermal fluctuations than those living at low or high altitudes in tropical regions (Rivera‐Rea et al., 2023; Zamora‐Camacho et al., 2016). In addition, other climatic factors, such as precipitation, can indirectly affect life‐history traits like survival, age structure, and sexual dimorphism by influencing food availability (Dunham, 1988). This phenomenon is commonly observed in lizards inhabiting cold climates, where the metabolic rate decreases due to low environmental temperatures, resulting in postponed growth and delayed age at maturity (Cabezas‐Cartes et al., 2015). Furthermore, seasonality is strong at high latitudes and has been shown to have favorable effects on survival rates by shortening annual activity periods (Adolph & Porter, 1993). Ecological factors, including predation pressure, can exert influence on the age at maturity as well (Scharf et al., 2015).

Animal body size variation is a complex phenomenon that exhibits both geographic and temporal dimensions (Green & Middleton, 2013). Hence, populations within species commonly exhibit geographic variations in body size (Altunışık, Üçeş, et al., 2022; Gaston et al., 2008; Tatlı et al., 2024). Besides, temporal changes in body size can manifest over both long and short time frames. For instance, evolutionary shifts can be seen on longer timescales (Millien et al., 2006), while shorter periods are marked by phenotypic plasticity, allowing for rapid alterations (Yom‐Tov & Geffen, 2006). Remarkably, significant shifts in mean body size have been observed over many years within various animal populations, including beetles (Braun et al., 2004), fishes (Ohshimo et al., 2009), and frogs (Tryjanowski et al., 2006). Sexual size dimorphism (SSD) is a common occurrence among reptiles (Cox et al., 2007; Olsson et al., 2002). Although fecundity selection and sexual selection theories help elucidate the range of variance in lizard SSD (Anderson & Vitt, 1990; Cruz‐Elizalde et al., 2020; Pincheira‐Donoso & Hunt, 2017), the influence of ecological and behavioral characteristics is also significant (Andersson, 1994; Hierlihy et al., 2013).

This study focuses on the orange‐tailed skink, *Eumeces schneiderii* (Daudin, 1802), which exhibits great ecological diversity across Türkiye. The species is listed as Least Concern by IUCN (Al Johany et al., 2021). As an ectothermic species, we questioned whether age structure, growth, body size, and sexual dimorphism of the orange‐tailed skink populations differ between two different environments/climates. Understanding the life‐history traits of this species can provide insight into the factors that contribute to sexual size dimorphism and longevity.

## 2 MATERIALS AND METHODS

### 2.1 Species and study sites

**FIGURE 1:**
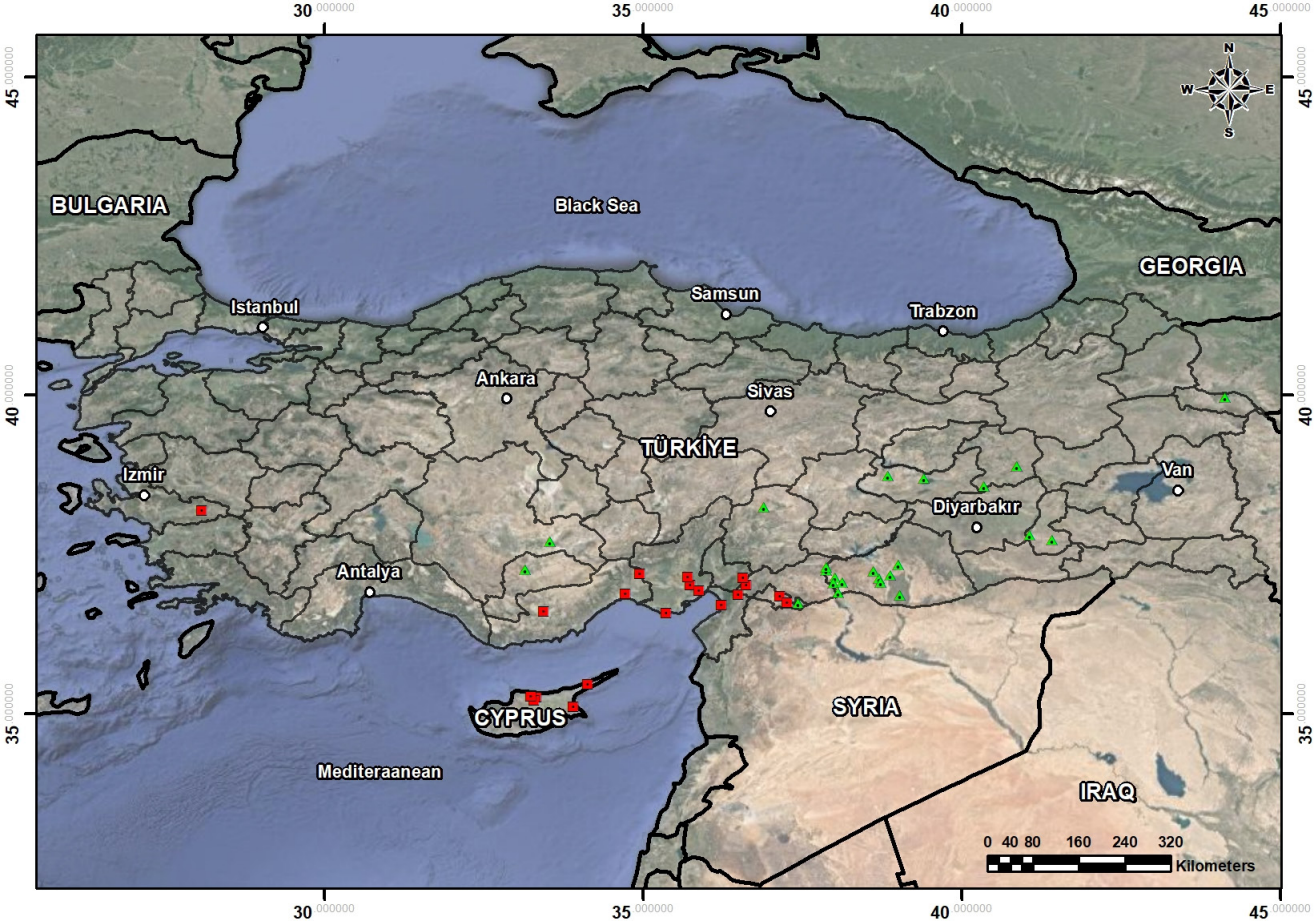
Sampling locations in this study. Red squares represent the distribution area of *Eumeces schneiderii* in Mediterranean sites and green triangles represent the distribution area of *Eumeces schneiderii* in continental sites.

A total of 88 *Eumeces schneiderii* specimens (16 males, 25 females, and 5 juveniles for Mediterranean sites; 20 males, 16 females, and 6 juveniles for continental sites) were acquired from Adıyaman University's Zoology Museum (ZMADYU), Türkiye (Figure 1). Sampling was performed with the permission of the Ege University Faculty of Medicine Animal Ethics Committee (Date: April 29, 2011, decision number: 2011/071). Sex was determined based on the presence or absence of a hemipenis (Altunışık et al., 2023). The most important color pattern feature that distinguishes juvenile individuals from adult individuals is the transverse or partially spotted white spots on the posterior edge of the dorsal scales. Also, these spots may form longitudinally dashed white lines along the body. Orange spots, which are found longitudinally in double rows or scattered in the dorsal region throughout the body in adult individuals, are indistinct in juveniles (Yalçınkaya, 2013). Specimens' SVL was measured using a digital caliper (Mitutoyo, Japan). Subsequently, following Smirina (1994), the fourth toe of the right hind limb was excised and preserved in a 70% ethanol solution.

### 2.2 Data collection and calculation

In Türkiye, the Mediterranean climate prevails across much of the Aegean Region, the western expanse of Central Anatolia, and the southern territories of the Taurus Mountains within the Mediterranean Region. Summers bring hot and dry weather, while winters are generally mild and marked by increased precipitation in this climate. Snowfall and frost are rarely seen in the coastal belt. Winters are snowy and cold in high areas (Sensoy et al., 2008). In this study, the average annual temperature in the locations with Mediterranean climates was 18.55°C (range: 17.3–19.3°C), while the lowest annual temperature average was recorded as 13.61°C (range: 11.9–14.1°C) (mgm.gov.tr). These regions experienced an average annual rainfall of 696.65 mm (range: 402.8–1157.8 mm) and the lizards were observed to be active from March to October.

In continental climate, the temperature difference between summer and winter is high, precipitation usually occurs in spring and winter, and drought dominates in summer. This climate prevails in the Central, Eastern, and Southeastern Anatolia regions and the inner part of Thrace (Sensoy et al., 2008). Mediterranean and continental zone populations are shown in Figure 1. In the study sites with continental climates, the average annual temperature was 14.21°C, with a range of 11.7°C to 18.5°C. Furthermore, the lowest recorded annual temperature averaged at 8.17°C, with variations from 5.4 to 12.7°C. These areas also experienced an average annual rainfall of 502.62 mm, ranging from 258.7 mm to 942.1 mm. Additionally, lizards were observed to be active from March–April to September in these sites.

### 2.3 Age determination

Skeletochronology is a commonly employed method for analyzing the age distribution of numerous ectothermic species. It relies on the observation of indicators called “growth markers” or “Lines of Arrested Growth” (LAGs), which develop in different types of bones, including phalanges, femurs, tibias, and humerus. The emergence of these markers is a result of decreased metabolic activity within bone tissue during estivation or hibernation (Gibbons & McCarty, 1983).

To conduct the skeletochronological investigation, modified techniques by Altunışık, Yıldız, et al. (2022) were employed, following Smirina's (1994) methods. The second phalanx, which was preserved in 70% ethanol, was first soaked in distilled water for a day, and then subjected to a decalcification process for approximately 2 h using a 5% HNO_3_. After obtaining cross‐sections with an 18 μm thickness using a Shandon cryostat microtome, Ehrlich's hematoxylin dye was applied to them and left for 10 min.

**FIGURE 2:**
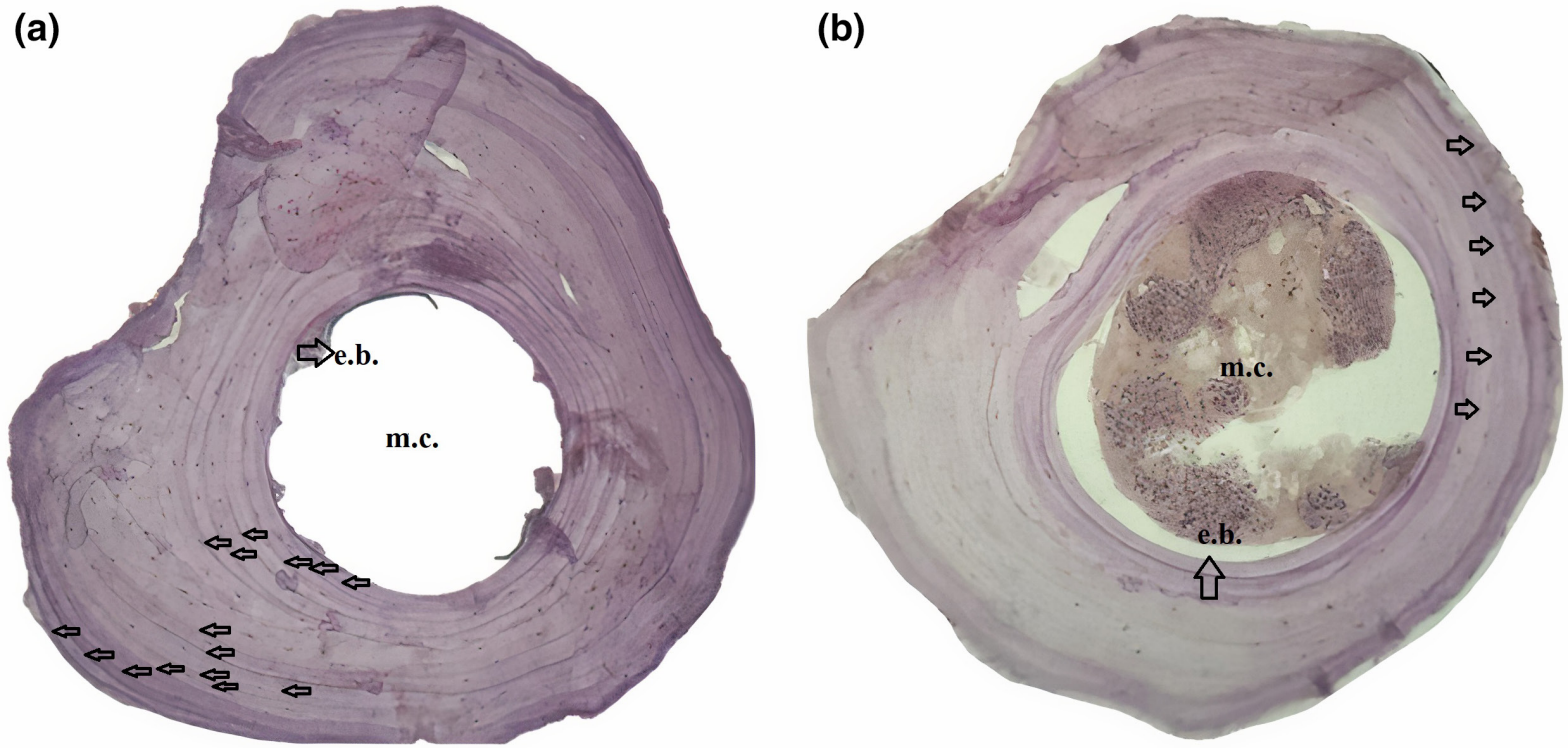
Cross‐sections (18 μm thick) at the diaphysis level of the phalange bone of *Eumeces schneiderii* specimens at the ages of 15 (a) and 6 (b). m.c., marrow cavity; e.b., endosteal bone.

Sections exhibiting rather narrow bone marrow cavities were specifically chosen and immersed in a glycerine solution. Using a light microscope equipped with a Pixera digital camera at 10× and 20× magnifications, the selected samples were observed and photographed (Figure 2). The authors conducted an independent count and confirmation of the LAGs subsequent to reviewing all captured images (Altunışık, Yıldız, et al., 2022).

### 2.4 Statistic and software

The experimental data underwent statistical analysis utilizing SPSS version 22.00. The data's normality was assessed via a Shapiro–Wilk test. For comparing sexes or populations, the parametric independent sample *t*‐test was employed. Pearson's correlation coefficient was used to investigate the association between SVL and age. Sexual size differences were estimated using the sexual dimorphism index (SDI) formulated by Lovich and Gibbons (1992).

(1) $$\mathrm{SDI}=\left(\frac{\text{size of larger}\;\mathrm{sex}}{\text{size of smaller}\;\mathrm{sex}}\right)$$

The methodology developed by Robson and Chapman (1961) was applied to calculate the survival rates (Svr).

$$\mathrm{Svr}=\frac{T}{\left(R+T–1\right)}$$

The calculation involves the use of an assumed constant survival rate, denoted as Svr in the formula (*T* = *n*₁ + 2*n*₂ + 3*n*₃ + 4*n*₄…, *R* = Σ ns, and ns = number of specimens in the age groups). This calculation accounts for the constrained annual survival rate. The 95% confidence intervals (CI) for survival rates were calculated as CI = 1.96 (*pq* − 1)^1/2^, where *q* = (1 − *p*); *p* is the survival rate; and *n* is the simple size.

The distance between two adjoining LAGs is a reliable indicator of individual growth over a given year. Where a clear decrease in spacing between two subsequent LAGs was observed, this was taken to mark the age at which sexual maturity was achieved (Kalayci et al., 2018; Ryser, 1988). To calculate the adult life expectancy (ESP), which signifies the expected lifespan of animals attaining sexual maturity, Seber's (1973) method was applied.

$$\mathrm{ESP}=0.5+\frac{1}{1-\mathrm{Svr}}$$

As in previous studies (Altunışık, Üçeş, & Yıldız, 2022; Guarino et al., 2010), the von Bertalanffy growth model was utilized to determine growth patterns and can be expressed generally as follows:

$$\text{SVLc}=\text{SVLmax}\;\left(1-e^{-k\left(c-t0\right)}\right).$$

In this formula, “SVLc” signifies size at age c, “SVLmax” represents the highest asymptotic SVL, “*e*” denotes the Euler's number (2.718), “*k*” defines the growth coefficient shaping the curve, and “*t*_0_” stands for the metamorphosis age. As the hatching size of *E. schneiderii* is not available, we adopted the size at hatching (SVLt_0_ = 25 mm) for other *Eumeces* species as indicated by Griffith (1991).

We employed MS Excel to compute SVLmax, *k*, and growth rates using “*r* = *k* (SVLmax − SVLt)” formula. To investigate variations in growth rates both within and between populations, we conducted *t*‐tests.

## 3 RESULTS

### 3.1 Demographic parameters and body size in the Mediterranean populations

**FIGURE 3:**
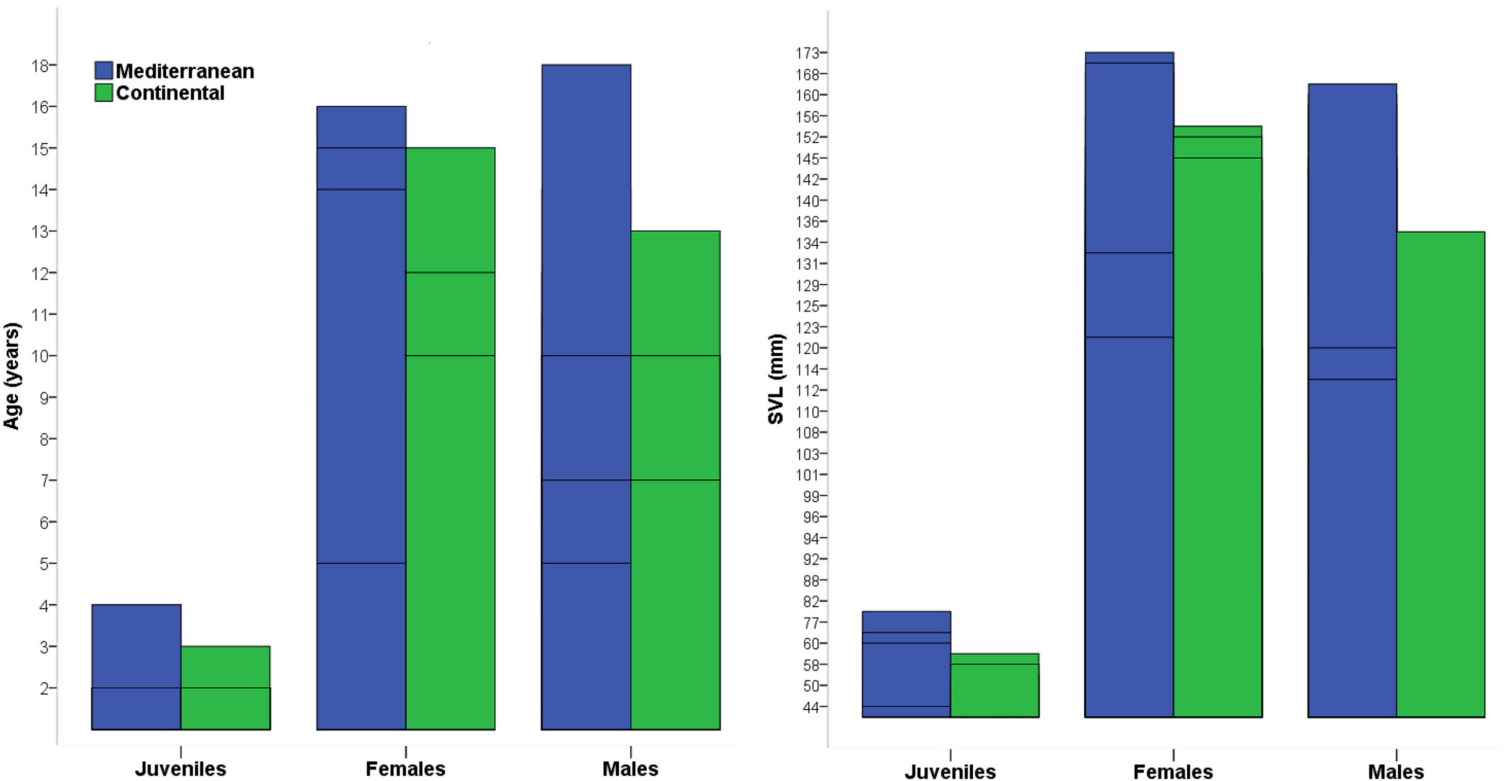
Age distribution graphic of *Eumeces schneiderii* from different sites.

**FIGURE 4:**
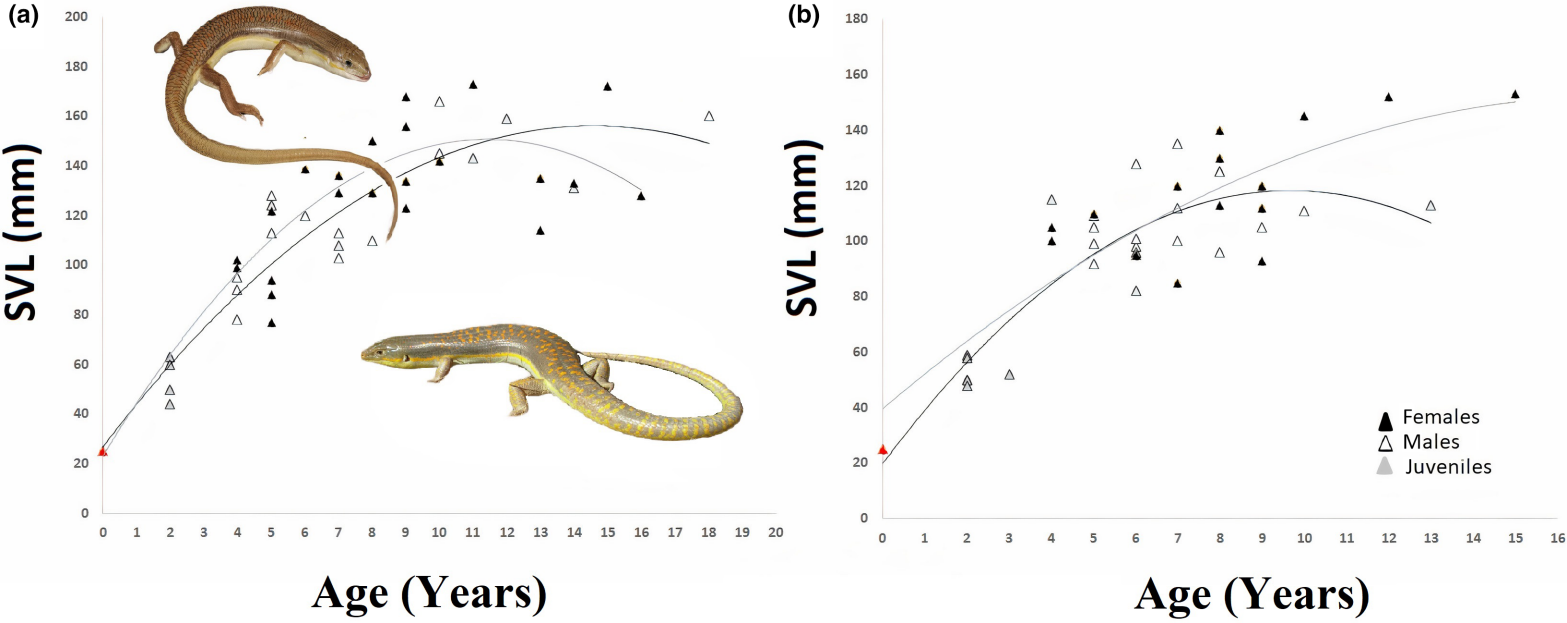
Relationship between age and body size (SVL) of *Eumeces schneiderii* from Mediterranean sites (a) and continental sites (b).

In Mediterranean group, the age varied from 4 to 18 years in male specimens (average: 8.31 ± 3.94) and 4 to 16 years in females (average: 8.52 ± 3.46) (Table 1). There was no statistical difference in the average age between males and females within this group (*t*‐test, *t* = −0.177, df = 39, *p* = .86). The 5th age class stands out as the most dominant in this site, comprising 17.07% (*n* = 7; see Figure 3). Sexual maturity was reached at 4 years for breeding individuals. The expected survival post‐maturity (ESP) for skinks attaining sexual maturity was computed as 7.10 (%95 CI: 1.97) years for males and 7.25 (%95 CI: 1.46) years for females. Both females and males demonstrated a Svr of 0.85 (%95 CI: female = 0.026; male = 0.033) (Table 2), signifying an 85% survival rate from 1 year to the later years. Growth rates were comparable between males (average: 4.20 ± 2.72 mm per year) and females (average: 2.90 ± 2.77 mm per year) within this population (*t*‐test, *t* = 0.468, df = 12, *p* = .648). The computed asymptotic SVL (SVLmax, males: 144.78 mm; females: 135.70 mm, Table 2) was lower than the maximum SVL recorded in this population for both males and females (Table 1). The growth parameters predicted using the von Bertalanffy equation indicate a fit that reflects the relationship between age and SVL (Figure 4a).

**TABLE 1:** Body size (SVL), longevity, and median age in some representative skink species and references.

Species	Location	Mean or range SVL (mm)		Mean or range age (years)		Longevity		Reference(s)
		Male	Female	Male	Female	Male	Female	
*Eulamprus leuraensis*	Southeastern Australia	52–84	66–86	2.25	3.00	6	6	Dubey et al., 2013
*Lamprolepis smaragdina*	Negros Island, Philippines	56.5		1–5		5		Alcala, 1966
*Plestiodon obsoletus*	Kansas, USA	70–130		6.2		8		Hall & Fitch, 1971
*Carinascincus ocellatus*	Tasmania, Australia	62.65–82.59	64.57–88.94	1–12		12		Wapstra et al., 2001
*Bellatorias major*	Eastern Australia	300–330		11–23		23		Chapple, 2003
*Chalcides ocellatus*	Western and Southern Türkiye	79.2	82.1	6.0	5.8	10	10	Mermer et al., 2020
*Ablepharus budaki*	Northwestern Türkiye	34.24		4.83		–	–	Yıldırım et al., 2017
*Eumeces schneiderii*	Mediterranean populations, Türkiye	125.50	131.08	8.31	8.52	18	16	This study
*Eumeces schneiderii*	Continental populations, Türkiye	107.10	118.31	6.85	8.13	13	15	

**TABLE 2:** Descriptive statistics of growth rate (mm per year), growth coefficient (k), ESP, and Sr in the studied populations of *Eumeces schneiderii* adults from Türkiye.

Group	Sex	N	Growth rate ± SE	k	SVLmax	ESP	Svr	SDI
Mediterranean	Males	16	4.20 ± 2.72	0.36	144.78	7.10	0.85	0.04
	Females	25	2.90 ± 2.77	0.51	135.70	7.25	0.85	
	Total	41	3.08 ± 2.84	0.85	142.69	7.07	0.85	
Continental	Males	20	3.78 ± 2.08	0.51	122.11	5.55	0.80	0.10
	Females	16	3.03 ± 2.81	0.29	143.78	6.96	0.84	
	Total	36	3.38 ± 3.03	0.44	127.31	6.04	0.82	

Abbreviations: ESP, adult life expectancy; *N*, number of specimens; SDI, sexual dimorphism index; SE, standard error of the mean; Svr, survival rate.

While females (mean: 131.08 mm) exhibited slightly larger sizes compared to males (mean: 125.50 mm), there was no substantial difference between the sexes (*t*‐test, *t* = −0.712, df = 39, *p* = .481). This finding was corroborated by the SDI calculated as 0.04 (see Table 2).

### 3.2 Demographic parameters and body size in the continental populations

The continental group consisted of specimens aged 4–13 years in males (mean: 6.85 ± 2.08) and 4–15 years in females (mean: 8.13 ± 2.82). The average age distinction between males and females was not statistically significant (*t*‐test, *t* = −1.558, df = 34, *p* = .128). The most prevalent age class in the population was the 6th age class, comprising 16.66% of the group (*n* = 7; Figure 3). Breeding individuals in this group attained sexual maturity at the age of 4 years. ESP was calculated to be 5.55 (%95 CI: 1.25) years for males and 6.96 (%95 CI: 1.69) years for females (Table 2). The survival rate (Svr) was calculated to be 0.80 (%95 CI: 0.037) for males and 0.84 (%95 CI: 0.035) for females, indicating that on average population's 82% survive from 1 year to later year. The calculated asymptotic SVL (SVLmax) was 144.78 mm for males and 135.70 mm for females (Table 2), which were found to be lower than the maximum SVL documented in this population for both sexes (Table 1). Analysis of growth parameters using the von Bertalanffy equation indicates a robust correlation between age and SVL, as depicted in Figure 4b.

Regarding growth rates, males displayed an average growth of 3.78 ± 2.08 mm per year, while females exhibited an average growth of 3.03 ± 2.81 mm per year. However, the distinction in growth rates between the males and females was not statistically significant (*t*‐test, *t* = 0.389, df = 15, *p* = .703). Similarly, there was no meaningful disparity in SVL between females (mean: 118.31 mm) and males (mean: 107.10 mm) (*t*‐test, *t* = −1.939, df = 34, *p* = .061), supported by an SDI of 0.10 (Table 2).

### 3.3 Comparison of the Mediterranean and continental populations

Our findings showed that the average body size of all specimens living in the Mediterranean region was higher than those in the continental region (*t*‐test, *t* = −2.797, df = 86, *p* < .01). According to the sex‐based evaluation, only body size of males differed between the two sites (*t*‐test, males: *t* = 2.950, df = 34, *p* < .01; females: *t* = 1.690, df = 39, *p* = .10). Regarding average age, there was no discernible difference between the two sites (*t*‐test, *t* = −1.498, df = 86, *p* = .138). When considering all populations collectively, it has been discovered that the species' maximum lifespan is 18 years (Table 1).

In females from both Mediterranean and continental populations, body size notably increased with age (Mediterranean: *r* = 0.488, *p* < .05; continental: *r* = 0.711, *p* < .01). However, among males, body size exhibited a positive increase with age solely within the Mediterranean group (*r* = 0.753, *p* < .01). Irrespective of gender, SVL also showed an increase with age in both populations (Mediterranean: *r* = 0.709, *p* < .001; continental: *r* = 0.767, *p* < .001). Through the utilization of the von Bertalanffy equation, the anticipated growth parameters portrayed a highly accurate connection between age and SVL (Figure 4). The growth rates of male and female orange‐tailed skinks living in different climates were similar (males: *t*‐test, *t* = 1.641, df = 12, *p* = .127; females: *t*‐test, *t* = 0.112, df = 12, *p* = .913). Statistical analysis showed the same result for all individuals, regardless of sex (*t*‐test, *t* = −1.486, df = 20, *p* = .153). The asymptotic size of male (144.78 vs. 122.11 mm) skinks living in the Mediterranean climate was found to be higher than those living in the continental climate. However, the asymptotic size calculated in females (143.78 vs. 135.70) was higher in the continental climate than in the Mediterranean climate sites (Table 2). On the other hand, ESP (7.07 vs. 6.04 years) and Svr (0.85 vs. 0.82) values were found to be higher in populations with Mediterranean climates compared to continental climates populations. Moreover, although there is a 10% disparity in size between male and female individuals in continental sites, this difference is restricted to 4% in Mediterranean sites (Table 2).

## 4 DISCUSSION

This pioneering study focused on several demographic factors including age, growth, survival rate, and the adult life expectancy of the orange‐tailed skink, living in different climates. Furthermore, a comparative analysis involving species of the Scincidae family (as detailed in Table 1) was executed concerning the mentioned parameters. The diversity in the evolution of life histories is influenced by two primary factors: environmental conditions (including resources like food and space, as well as temperature, Roff, 1992, Stearns, 1992) and genetic factors (as explored by Ballinger (1979) and Dunham and Miles (1985)). Notably, substantial variations exist in the life histories of lizard populations, sex, and across distinct natural settings, even within the confines of the same genus or species. In this context, the analysis of orange‐tailed skink populations of the same species living in different climates indicates that there is no statistical difference in mean age. Upon closer inspection of Table 1, it becomes evident that species within the same family, residing in diverse ecological settings, exhibit distinct life‐history traits. Males and females of *E. schneiderii* analyzed in this study were estimated to live up to a maximum of 18 years, which is one of the highest values among wild skink populations. For example, it has been reported that *Bellatorias major*, which has a relatively large body of 33 cm, lives up to 23 years old at most (Chapple, 2003). On the other hand, *Lamprolepis smaragdina* has a short lifespan of 5 years (Alcala, 1966). The longevity of those skink species, which inhabit diverse geographical regions, might have been impacted by local environmental conditions, the abundance of food, and their genetic makeup.

The orange‐tailed skinks reached sexual maturity at a mean age of 4 years in both Mediterranean and terrestrial populations. The similar age at sexual maturity in both regions may be related to growth and age structure, as the means of these two traits were not statistically different between the two regions. Additionally, the fact that the active period of the orange‐tailed skinks living in the Mediterranean climate is only 1 month longer than those living in a continental climate can be considered among the reasons for this similarity.

The Svr of the Mediterranean and continental populations exhibited distinct tendency in male and female specimens in the present investigation. For instance, whereas the Svr of females (0.84) was higher compared to males (0.80) in the continental populations, this rate was same in both sexes (0.85) in the Mediterranean populations (Table 2). Similar to the survival rates, sex variation in adult life expectancy in the continental populations is more pronounced than in the Mediterranean populations. On the other hand, low survival rates in the Blue Mountains water skink have been reported as an effective factor for the species to be categorized as endangered (Dubey et al., 2013). When analyzing the growth rates, it is clear that the Mediterranean population and the terrestrial population have similar characteristics. Previous research found that the *Nivoscincus ocellatus* population exhibits a higher growth rate in warm regions compared to cold regions (Wapstra et al., 2001). Additionally, Mermer et al. (2020) suggested that the growth rate of the western Mediterranean population of *C. ocellatus* is slower than that of the eastern Mediterranean population, with climate likely playing a role in this disparity. James (1991), on the other hand, investigating life‐history traits in five scincid lizards of the genus *Ctenotus*, reported that females of these species grew more slowly but reached a larger asymptotic body size. A similar result was obtained for the continental populations in this study, with females having a larger asymptotic body size and lower growth rate (Table 2).

When evaluating all individuals, regardless of sex, it was found that lizards living in habitats with a Mediterranean climate had larger body sizes than those living in continental climates. This result is an exception to the hypothesis that “individuals of the same species living in colder regions have larger bodies than those living in warmer regions,” and is not common in the literature (Ashton & Feldman, 2003; Oufiero et al., 2011). The variance in the results could possibly be due to differences in the activation period between the regions (Horváthová et al., 2013). The Mediterranean region, with its longer activation periods and milder, wetter climate compared to the continental region, is likely to have provided more favorable conditions for growth. Alternatively, another contributing factor could be the abundance of food resources (personal observations from the field studies), which may favor Mediterranean populations. The difference in growth is often regarded as one of the main reasons for the sexual size dimorphism. Trade‐offs between energy allocation to growth and reproduction, which vary across species and between sexes, contribute to the divergence in growth patterns, potentially giving rise to differing SSD patterns (Yang et al., 2019). Exploring the underlying reasons for SSD yields insights into the intricate interplay between ecological factors and evolutionary forces. Notable studies by Cox et al. (2007) and Roitberg (2007) highlight the potential drivers behind divergent SSD trends, both within and among species. These differences are often explained by basic hypotheses such as sexual selection, natural selection, and fecundity selection (Altunışık, 2017; Andersson, 1994). For example, as described by Fairbairn (1997), rivalry for food between animals can contribute to sexual dimorphism in various ways. In this study, body size difference in both Mediterranean and continental orange‐tailed skink populations is female biased, albeit weakly. This female‐biased phenomenon has also been documented in small‐sized species such as *Lacerta viridis* (Altunışık et al., 2023), *L. agilis* (Guarino et al., 2010), *Cyrtopodion scabrum* (Altunışık, Üçeş, & Yıldız, 2022), *Chalcides ocellatus* (Mermer et al., 2020), and *C. chalcides* (Guarino, 2010).

In studies concentrating on the life‐history characteristics of ectothermic organisms, one parameter that is frequently explored is the association between age and body size. While a positive and significant relationship between these two parameters was observed in certain species (*Chalcides chalcides*: Guarino, 2010; *Asaccus barani*: Kalaycı et al., 2015; *Chalcides ocellatus*: Mermer et al., 2020; *Mediodactylus heterocercus*: Altunışık, Yıldız, et al., 2022), it has been reported that no significant correlation exists in some other species such as *Macroscincus cocte* (Andersson & Guarino, 2003), *Eulamprus leuraensis* (Dubey et al., 2013), and *Phoenicolacerta laevis* (Bülbül et al., 2021). In line with the overall trend, the body size of orange‐tailed skinks significantly grows with age in both Mediterranean and continental populations.

## 5 CONCLUSION

Our initial findings, highlighting insights into the longevity, age structure, age at sexual maturity, body size, growth, survival rates, and adult life expectancy of the orange‐tailed skink populations residing in diverse climates across Türkiye, are instrumental in advancing our ecological understanding of these lizard species. In conclusion, lizards living in habitats characterized by milder Mediterranean climates were found to have larger body sizes than continental populations, but both populations were similar in terms of mean age. The age structure appears to be compatible with the growth parameters of the orange‐tailed skinks living in different climates. However, the reason for the larger size found in Mediterranean populations may be related to several factors, including activation time, temperature, precipitation, food abundance, and the presence of predators.

## AUTHOR CONTRIBUTIONS

**Abdullah Altunışık:** Formal analysis (equal); investigation (equal); methodology (equal); supervision (equal); visualization (equal); writing – original draft (equal); writing – review and editing (equal). **Mehmet Zülfü Yıldız:** Conceptualization (equal); data curation (equal); funding acquisition (equal); investigation (equal); project administration (equal); resources (equal); supervision (equal); validation (equal); writing – review and editing (equal). **Hatice Hale Tatlı:** Conceptualization (equal); formal analysis (equal); investigation (equal); methodology (equal); software (equal). **Deniz Yalçınkaya:** Conceptualization (equal); data curation (equal); funding acquisition (equal); project administration (equal); resources (equal); software (equal). **Bahadır Akman:** Conceptualization (equal); data curation (equal); formal analysis (equal); investigation (equal); project administration (equal); resources (equal); visualization (equal).

## FUNDING INFORMATION

This research received no specific grant from any funding agency in the public, commercial, or not‐for‐profit sector.

## CONFLICT OF INTEREST STATEMENT

The authors declare that they have no known competing financial interests or personal relationships that could have appeared to influence the work reported in this paper.

## Supporting information


Data S1:

## Data Availability

All data generated or analyzed during this study are included in this manuscript and supplementary file.
